# Adult Deletion of SRF Increases Epileptogenesis and Decreases Activity-Induced Gene Expression

**DOI:** 10.1007/s12035-014-9089-7

**Published:** 2015-01-31

**Authors:** Bozena Kuzniewska, Karolina Nader, Michal Dabrowski, Leszek Kaczmarek, Katarzyna Kalita

**Affiliations:** 1 Laboratory of Neurobiology, Nencki Institute, 3 Pasteur Street, Warsaw, Poland; 2 Laboratory of Bioinformatics, Neurobiology Center, Nencki Institute, 3 Pasteur Street, Warsaw, Poland

**Keywords:** SRF, Epilepsy, Gene expression, Hippocampus, Plasticity

## Abstract

Although the transcription factor serum response factor (SRF) has been suggested to play a role in activity-dependent gene expression and mediate plasticity-associated structural changes in the hippocampus, no unequivocal evidence has been provided for its role in brain pathology, such as epilepsy. A genome-wide program of activity-induced genes that are regulated by SRF also remains unknown. In the present study, we show that the inducible and conditional deletion of SRF in the adult mouse hippocampus increases the epileptic phenotype in the kainic acid model of epilepsy, reflected by more severe and frequent seizures. Moreover, we observe a robust decrease in activity-induced gene transcription in SRF knockout mice. We characterize the genetic program controlled by SRF in neurons and using functional annotation, we find that SRF target genes are associated with synaptic plasticity and epilepsy. Several of these SRF targets function as regulators of inhibitory or excitatory balance and the structural plasticity of neurons. Interestingly, mutations in those SRF targets have found to be associated with such human neuropsychiatric disorders, as autism and intellectual disability. We also identify novel direct SRF targets in hippocampus: *Npas4*, *Gadd45g*, and *Zfp36*. Altogether, our data indicate that proteins that are highly upregulated by neuronal stimulation, identified in the present study as SRF targets, may function as endogenous protectors against overactivation. Thus, the lack of these effector proteins in SRF knockout animals may lead to uncontrolled excitation and eventually epilepsy.

**Electronic supplementary material:**

The online version of this article (doi:10.1007/s12035-014-9089-7) contains supplementary material, which is available to authorized users.

## Introduction

Epilepsy is a chronic neurological disorder that affects approximately 1 % of the human population [1]. The development of epilepsy (epileptogenesis) involves progressive alterations in synaptic connections (aberrant plasticity), the molecular mechanisms of which are still poorly understood. The regulation of gene transcription by neuronal activity is an integral part of adaptive plasticity [2]. Therefore, the identification of gene expression programs that result from and control long-term synaptic changes is crucial for understanding the molecular mechanisms that underlie epilepsy [3].

Serum response factor (SRF) is a MADS-box protein that binds DNA at the CC(A/T)_6_GG consensus sequence, known as a CArG box or serum response element (SRE). SREs are found in promoters of actin cytoskeleton genes and immediate-early genes (IEGs; [4–6]). SRF-dependent transcription is activated by neurotrophins or in response to calcium influx in neurons [7–11]. The inactivation of SRF leads to a decrease in the expression of such plasticity-linked genes as *Fos*, *Arc*, and *Egr1* and produces a deficiency in hippocampal synaptic plasticity and learning [12, 13]. The activity of SRF is also important during the development of the nervous system [14–16] and the regulation of structural plasticity [17, 18]. The molecules that are involved in physiological plasticity, such as SRF, may also be engaged in pathological or aberrant plasticity processes.

Although SRF has recently been suggested to play a role in plasticity and mediate structural changes in the hippocampus, no solid evidence has been provided for its role in brain pathology. Interestingly, the increased binding of SRF to DNA and upregulation of SRF protein levels were found in the hippocampus after pilocarpine-induced status epilepticus, and SRF accumulation and phosphorylation were observed after kainic acid-induced status epilepticus [19, 20]. These findings indicate that the transcriptional activity of SRF is enhanced during epileptogenesis but provide no explanation for its precise role in this brain pathology. Furthermore, many SRF-dependent genes that are important for synaptic plasticity likely still remain unidentified, and no global analysis of SRF-dependent gene expression in response to neuronal stimulation in the adult brain has yet been reported.

In the present study, we investigated the role of the SRF-dependent transcriptional program in epileptogenesis using brain-specific, inducible SRF gene knockout (KO) in mice. We found that SRF KO mice exhibited an increase in the susceptibility to spontaneous seizure development and more severe seizures. We also identified 378 activity-dependent SRF target genes, among which we distinguished a group with functions associated with epilepsy and synaptic plasticity that may be responsible for the observed phenotype. Furthermore, we identified several novel genes that are directly regulated by SRF in the hippocampus in vivo: *Npas4*, *Gadd45g*, and *Zfp36*.

## Materials and Methods

### Animals

Mice with the conditional deletion of *Srf* in forebrain neurons (*Srf* ^*CaMKCreERT2*^; mutant mice; KO) were generated by crossing the *Srf* ^f/f^ mutant strain (stock nr: 006658; Jackson’s Laboratories, USA; [12]), carrying the loxP flanked *Srf*, with mice expressing Cre recombinase under the control of neuron-specific CaMKIIα promoter which is activated only in excitatory forebrain neurons (strain nr: EM:02125; The European Mouse Mutant Archive; [21]). To obtain time-specific control of the genetic process, Cre recombinase was originally fused with mutated ligand-binding domain of human estrogen receptor to obtain CreERT2 enzyme which is translocated to the nucleus and active only in response to tamoxifen (TAM) treatment. CreERT2-negative littermates were used as a control (*Srf* ^f/f^; control mice; CTR). Adult mice, both genotypes, CTR and KO (2–6 months) were injected intraperitoneally with 1 mg of TAM twice daily for 5–10 days (Sigma). Experiments were performed at least 4 weeks after tamoxifen injections. For the ChIP experiments adult C57BL/6 mice were used. The animals were treated in accordance with the ethical standards of European and Polish regulations. All experimental procedures were approved by the Local Ethics Committee. The mice were bred in the Animal House of Nencki Institute of Experimental Biology, Warsaw. Animals were housed in individual cages under 12-h light/dark cycle with constant temperature (23–25 °C) and with food and water available ad libitum.

### Immunohistochemistry

Mice were transcardially perfused with 4 % paraformaldehyde in PBS. Brains were further fixed in the same buffer overnight at 4 °C and cut on vibratome on 40-μm slices. Free floating brain slices were permeabilized, blocked in normal goat serum for 1 h at room temperature, washed, and incubated with anti-SRF antibody (1:500) (H-300; Santa Cruz Biotechnology) overnight at 4 °C. Next, immunochistochemistry was performed using standard procedure with Avidin/Biotin Complex (ABC) Kit Vectastain PK-6100 (Vector Labs) and visualized using SIGMAFAST™ DAB (Sigma). For Nissl staining, brain sections were air dried on slides. Next, the slides were stained with 0.1 % cresyl violet solution (containing 3 % acetic acid) for 5 min, washed, dehydrated, cleared in xylene, and coverslipped.

### Western Blotting

Dentate gyrus of the hippocampus was dissected and homogenized in a lysis buffer containing 1 mM MgCl_2_, 5 mM HEPES (pH = 7.4), 320 mM sucrose, 1 mM Na_2_F, and protease inhibitor cocktail complete (Roche) using Dounce glass homogenizer. Protein concentration was measured using BCA Protein Assay Kit (Pierce). Fifteen microgram of homogenates was dissolved on 8 % polyacrylamide gels. Western blot was performed using a standard procedure using anti-SRF antibody (1:300) (H-300; Santa Cruz Biotechnology). Blots were reprobed with an anti-α-tubulin antibody (1:5000) (Sigma) to ensure equal total protein levels. Chemiluminescent detection method was used. For the quantification of individual bands, the scan of the photographic film was analyzed by densitometry using GeneTools software (Syngene).

### Kainic Acid Treatments

CTR and KO animals were injected with 70 nl of 20 mM solution of kainic acid (KA, Nanocs) in 0.9 % NaCl or saline at the left CA1 area of the dorsal hippocampus. For intrahippocampal injection of kainic acid only males were used. For intraperitoneal injections mice were habituated to handling and injected with saline for 3 days before the experiments. Then, CTR and SRF KO were injected intraperitoneally with 35–50 mg/kg kainic acid or saline. For the microarrays profiling experiments only male mice were used. Confirmation of microarray results by qRT-PCR was performed on males and females. Six hours after kainic acid-induced status epilepticus (SE) or 6 h after saline injection, mice were sacrificed and brain hemispheres were dissected and incubated overnight in RNAlater solution in 4 °C (Ambion). Next, the dentate gyrus of the hippocampus was dissected and frozen in −80 °C for further RNA isolation. For ChIP assay only males were used. Animals 2 h after kainic acid-induced status epilepticus were sacrificed and the hippocampus dissected and used for chromatin immunoprecipitation.

### Surgery and Video-Electroencephalography (EEG)

#### Surgical Procedures

Animals (adult males) were anesthetized by inhalation of 1.5–2 % isoflurane (Baxter) in oxygen and placed in a stereotaxic frame and on a heating pad in order to maintain a constant body temperature. Mice were injected with kainic acid (with rapidity of 50 nl/min) or saline (control animals) aimed at the left CA1 area of the dorsal hippocampus with following coordinates: AP −1.8 mm, ML +1.7 mm, and DV −2.3 mm from bregma.

#### Electrode Preparation, Implantation, Electroencephalography, and Video Recordings

Cortical electrodes were home-made: a screw was joined to socket contact by copper wire and welded (Bilaney Consultants Ltd.). Two electrodes were placed bilaterally into the scull over the frontal cortex and next two over the cerebellum (first as reference and second as ground electrode). Bipolar hippocampal electrode (Bilaney Consultants Ltd.) was implanted into the injected hippocampus using the coordinates AP −2.0 mm and ML +1.3 mm with bregma as reference point and DV −1.7 mm below dura. Electrodes were placed into pedestal (Bilaney Consultants Ltd.) and secured with dental acrylate (Duracryl Plus, Spofa Dental, Czech Republic). After operation, mice were connected to a digital acquisition system (TWin Clinical Software for EEG, Grass Technologies) and EEG activity of freely behaving animals was monitored for 18 days in an isolated room. The unit consisted of six cages.

#### Analysis of Video-EEG Monitoring

The occurrence of spontaneous seizures was studied by browsing through EEG record on a computer screen. All motion artifacts were excluded from analysis. An encephalographic seizure was specified as a high-amplitude (more than 2× baseline) discharge lasting more than 5 s. Several seizure parameters were characterized: behavioral severity (average score), seizure frequency, and average seizure duration. Behavioral severity was analyzed from matching video-EEG recording. Seizures were estimated based on modified Racine’s scale [22]: score 1—immobility, facial clonus; score 2—“wet-dog” shakes, head nodding; score 3—piano pose (bilateral clonus of forepaws); score 4—clonic seizures with falling; and score 5—generalized tonic-clonic seizures. Seizure frequency was specified as a total number of detected seizures divided by recording days, and duration was determined on the basis of EEG recordings. SE severity was analyzed during first 24 h after intrahippocampal injection of KA. Spontaneous seizures were defined as seizures appearing as early as 24 h after KA administration.

### Gene Expression Profiling Using Microarrays

Total RNA was isolated from the dentate gyrus using RNeasy Mini Kit (Qiagen) as described by the manufacturer. Residual DNA was removed by digestion with DNase I (Qiagen). RNA concentration was calculated from the absorbance at 260 nm. Purity and integrity of RNA was determined using Agilent RNA 6000 Nano Chips on the Agilent 2100 Bioanalyzer System (Agilent Technologies). A starting amount of 200-ng high-quality total RNA was used to generate complementary DNA (cDNA) and complementary RNA (cRNA) with the Illumina TotalPrep RNA Amplification Kit (Illumina Inc., San Diego, CA, USA). The obtained cDNA served as a template for in vitro transcription with T7 RNA polymerase and biotin UTP to generate multiple copies of biotinylated cRNA. Each cRNA sample (1.5 μg) was hybridized overnight to MouseWG-6 BeadChip array (Illumina); subsequently, chips were washed, dried, and scanned with the BeadArray Reader (Illumina). A total of 22 microarrays were used (5–6 biological replicates per group). Analysis and quality control of microarrays were performed using BeadArray R package. After background subtraction, data was normalized using quantile normalization and then log2-transformed. The obtained signal was taken as the measure of mRNA abundance derived from the level of gene expression. Statistical analysis of the microarray results was performed using a two-way ANOVA (for the factors genotype and treatment) followed by the estimation of FDR (percent FDR; false discovery rate) using the Benjamini and Hochberg method. Microarray data are available in the NCBI Gene Expression Omnibus (GEO) under accession number GEO: GSE60772.

Gene ontology enrichment analysis was performed using DAVID database. Pathway and global functional analysis was performed using Ingenuity Pathway Analysis (IPA; Ingenuity® Systems, Qiagen). Additionally, manual analysis of genes’ functions and their role in neurons was assigned on the basis of published data (manual search of PubMed).

### In Silico Analysis of Putative SRF-Binding Sites

Overrepresentation analysis of transcription factor binding sites (TFBS) in the group of identified genes was performed using the cREMaG database with default parameters [23].

Independently, using NGD database [24] we identified genes with at least two putative SRF-binding sites in the conserved noncoding sequences between mouse and human, identified with programs AVID-VISTA [25, 26] within the −10 kb/+10 kb from Transcription Start Site. Potential SRF-binding sites were predicted using all the motifs from the $SRFF family (Genomatix MatBase v. 8.4). Moreover, for the selected genes (Table 3) we compared previously identified potential CArG boxes in the human genome [27] to the mouse sequences. Among the putative SRF-binding sites identified using the three above approaches (cREMaG, NGD, CArG), for further analysis, we chose only the sites conforming exactly to the consensus sequence motif: CC(A/T)_6_GG or having a maximum two base pair mismatch (only one mismatch in the CC or GG) to the consensus sequence.

### RNA Preparation and Quantitative Real-Time PCR

Total RNA was isolated from the dentate gyrus of the hippocampus using RNeasy Mini Kit (Qiagen) as described by the manufacturer. Residual DNA was removed by digestion with DNase I (Qiagen). RNA concentration was calculated from the absorbance at 260 nm and the purity of RNA was determined by the 260/280 nm absorbance ratio. RNA was reverse transcribed with the SuperScript III Reverse Transcriptase (Invitrogen) according to the manufacturer’s instructions. Quantitative real-time PCR reactions were performed using Fast SYBR Green Master Mix (Applied Biosystems) in the Applied Biosystems 7900HT Fast Real-Time PCR System using the following cycling conditions: 50 °C for 2 min, 95 °C for 10 min, followed by 40 cycles of 95 °C for 15 s and 60 °C for 1 min. Primer sequences are presented in Table 1. Fold changes in expression were determined using the ∆∆C_T_ relative quantification method. Values were normalized to the relative amounts of ribosomal protein large P0 (Arbp) mRNA, which was found to be stable across different phases of KA-induced epilepsy in mice [28].

**Table 1:** List of primer sequences used for qRT-PCR validation of selected microarray results

Gene symbol	Primers sequences
*Arbp*	F: 5′ AGATTCGGGATATGCTGTTGGC 3′
	R: 5′ TCGGGTCCTAGACCAGTGTTC 3′
*Fos*	F: 5′ CCCATCCTTACGGACTCCC 3′
	R: 5′ GAGATAGCTGCTCTACTTTGCC 3′
*Npas4*	F: 5′ GCTATACTCAGAAGGTCCAGAAGGC 3′
	R: 5′ TCAGAGAATGAGGGTAGCACAGC 3′
*Bdnf*	F: 5′ CTGTAGTCGCCAAGGTGGAT 3′
	R: 5′ AGAAGTTCGGCTTTGCTCAG 3′
*Syt4*	F: 5′ TGACCCGTACATCAAAATGACAA 3′
	R: 5′ GTGGGGATAAGGGATTCCATAGA 3′
*Gadd45g*	F: 5′ GAAAGCACTGCACGAACTTCT 3′
	R: 5′ CTTTGGCGGACTCGTAGACG 3′
*Acan*	F: 5′ GTGGAGCCGTGTTTCCAAG 3′
	R: 5′ AGATGCTGTTGACTCGAACCT 3′
*Lcn2*	F: 5′ GGGAAATATGCACAGGTATCCTC 3′
	R: 5′ CATGGCGAACTGGTTGTAGTC 3′
*Pcdh8*	F: 5′ TTCCCCTTGCAGCTATTTAGTCT 3′
	R: 5′ GAACGTGCTGTATCGGACTGT 3′
*Elmo1*	F: 5′ GGAGAAGATCCAGCCAGAAAT 3′
	R: 5′ GCGAGCATTGAGTTTCCTAAAG 3′
*Magoh*	F: 5′ ACTTTTACCTGCGTTACTACGTG 3′
	R: 5′ GTTGTTGGCGTATCGCAATTT 3′
*Zfp36*	F: 5′ TCTCTGCCATCTACGAGAGCC 3′
	R: 5′ CCAGTCAGGCGAGAGGTGA 3′

### Chromatin Immunoprecipitation

C57BL/6 mice were injected with KA (35–50 mg/kg), and 2 h after KA-induced status epilepticus or saline injection, hippocampi were dissected, chopped (0.5-mm fragments), and fixed with 1.5 % formaldehyde for 30 min at room temperature, then glycine was added and incubated for 10 min to quench formaldehyde (hippocampi from each mouse were processed separately). Samples were put on ice, washed with ice-cold phosphate-buffered saline (PBS) supplemented with protease inhibitor cocktail (Roche) and 1 mM PMSF and briefly homogenized with an Eppendorf fitting pestle. Samples were centrifuged again, washed with PBS with protease inhibitors, and centrifuged (1.3 g/5 min/4 °C), and the pellet was resuspended in 2 ml of lysis buffer (5 mM PIPES, pH 8.0, 85 mM KCl, 0.5 % Nonidet P-40, with protease inhibitors), incubated for 10 min on ice, and disrupted using a glass–glass homogenizer. Crude nuclear extract was collected by centrifugation at 1.3 g for 10 min at 4 °C and frozen in −80 °C. Nuclear extracts were pooled and resuspended (3 hippocampi in 200 μl) in high-salt lysis buffer with protease inhibitors (1× PBS, 1 % Nonidet P-40, 0.5 % sodium deoxycholate; 1 % SDS). Samples were sonicated on ice 14 times at 25 % output for 20 s with 30 s breaks (on ice) using a Branson Sonifier 250 obtaining an average of 300–500 bp DNA fragments. Samples were diluted 10× with high-salt lysis buffer without SDS (final concentration of 0.1 % SDS), centrifuged for 15 min to remove cell debris, and precleared with sonicated salmon sperm DNA/protein A agarose (50 % slurry) at 4 °C for 30 min with rotation. The DNA content was measured spectrophotometrically, and equal amounts of DNA were immunoprecipitated overnight at 4 °C using 5-μg anti-SRF antibody (H-300, Santa Cruz) or 5 μg of normal rabbit IgG as negative control. Next day, salmon sperm DNA protein A-agarose beads (Milipore) were added for 2 h, then the beads were washed with low-salt wash buffer (0.1 % SDS, 1 % Triton X-100, 2 mM EDTA, 20 mM Tris–HCl, pH 8.1, 150 mM NaCl), high-salt wash buffer (0.1 % SDS, 1 % Triton X-100, 2 mM EDTA, 20 mM Tris–HCl, pH 8.1, 500 mM NaCl), LiCl wash buffer (0.25 M LiCl, 1 % Nonidet P-40, 1 % deoxycholate, 1 mM EDTA, 10 mM Tris–HCl, pH 8.1), and twice in TE. Antibody-DNA complexes were eluted with elution buffer (1 % SDS, 0.1 M NaHCO_3_), crosslinking was reversed, and proteins were removed by adding 5 M NaCl and proteinase K and incubation for 2 h at 42 °C, followed by incubation at 65 °C overnight, next DNA was purified using QIAquick Gel Extraction Kit (Qiagen). Immunoprecipitated chromatin was used as a template for real-time PCR reactions using Fast SYBR Green Master Mix (Applied Biosystems) in the Applied Biosystems 7900HT Fast Real-Time PCR System with specific primers located near the predicted SRF binding sites (Table 2). Obtained data was analyzed using the ∆∆C_T_ relative quantification method and calculated as the percentage of the input.

**Table 2:** List of putative SRF binding sites within the evolutionary conserved regions between mouse and human (10/+10 kb from transcription start site [TSS]) that were selected for experimental validation. Mismatched nucleotides (compared with SRF consensus motif) are underlined. The primer sequences used in the ChIP assays and location of amplicons (relative to TSS) are indicated. The results of the ChIP analysis (see also Fig. 5) are presented

Gene symbol	Selected predicted binding site position	Predicted CArG box sequence	Amplicon location	Primers sequences	SRF-binding
*Fos*	−309/−300	CCATATTAGG	−391/−260	F: 5′ TCCCCCCCTGCGCTGCACCCTCAGA 3′	*Y*
				R: 5′ CAACAGGGACCGGCCGTGGAAACCT 3′	
*Npas4*	−960/−951	CCTAATATGG	−1130/−1033	F: 5′ AAAGGGTCTTGGGTAGGTGC 3′	*Y*
				R: 5′ CCTCCGCACAGCTCTAGAAA 3′	
	−4109/−4100	CCAAATATGG	−4207/−4032	F: 5′ TCAGTTGTGTGTGTGCCTGT 3′	*Y*
				R: 5′ GAGCACCCTTCTCTGGAACC 3′	
*Gadd45g*	−31/−22	CTATAAAAGG	−177/−86	F: 5′ TTTGAGGCTGTGTCATCCCC 3′	*Y*
	−263/−254	CCTTTTAAGG		R: 5′ GCCCGCTTTCTGATGCAAAT 3′	
*Lcn2*	−1674/−1665	CCTTTTAAGG	−1728/−1605	F: 5′ GTCTCCCATGTGCTGGATAAA 3′	N
				R: 5′ GTGACCTCTCCACCCTTCTA 3′	
*Syt4*	−1502/−1493	CCATATGAAG	−1623/−1542	F: 5′ TCAACTCTGGGCAATCGGTC 3′	N
				R: 5′ TTTCTCCAAAGCTCAGCCCA 3′	
*Bdnf*	+4620/+4629	CCAGAAATGG	+4418/+4334	F: 5′ GGGAGGAAGAGAGGGAGAGA 3′	N
				R: 5′ GCAAGACCAGGGGCTACAAA 3′	
	−9372/−9363	CCCATTCTGG/CCATTCTGGG	−9319/−9154	F: 5′ ACCCCATCTTTGATTTGCAGC 3′	N
				R: 5′ GCCCCAGTTGCCCTACATTT 3′	
	−9336/−9327	CCTAATTTGC			N
*Magoh*	−2982/−2973	CCACATGTGG	−3026/−2876	F: 5′ GCTGAAGGAAGTGTGCTCCA 3′	N
	−2441/−2432	CTTATTTTGG		R: 5′ CCTCCCCACCACCATCAAAT 3′	
*Zfp36*	+3473/+3482	CCATACAAGG	+3670/+3867	F: 5′ CCCTCTGTCTCTTAGCCCCT 3′	*Y*
				R: 5′ TCACAAGGGAGGCAGTTTCC 3′	
*Pcdh8*	−1061/−1052	TCTAAATAGG	−1151/−1035	F: 5′ CAGGTTTCAACGTCACGCTG 3′	N
	−1086/−1077	CCCTATTAGA			
				R: 5′ CTGATGCCTTGTTCCGCCTA 3′	
*Elmo1*	+8069/+8078	CCAATTATGG	+7998/+7879	F: 5′ ATGTGGCTTGGGAGGTTGAG 3′	N
				R: 5′ GTGGGCAGGAGTCAAGTTGA 3′	
*Arbp exon*	–	–	+1509/+1712	F: 5′ AGATTCGGGATATGCTGTTGGC 3′	
				R: 5′ TCGGGTCCTAGACCAGTGTTC 3′	–

*Y* yes (SRF binding observed), *N* no (no SRF binding in the analyzed promoter region detected)

### Statistical Analysis

Data on the graphs are expressed as means ± standard errors of the means (SEM) from at least three independent experiments. The statistical analysis of the data was performed with GraphPad Prism software (GraphPad Software, Inc.). For comparison of two groups either unpaired *t* test or Mann–Whitney test (nonparametric) was used. For comparison of multiple groups, a two-way analysis of variance (ANOVA) with post hoc Bonferroni’s multiple comparisons test was used.

## Results

### Characterization of *Srf*^f/f;CaMKCreERT2^ Mice

To investigate the role of SRF in the development of temporal lobe epilepsy (TLE), *Srf* gene KO mice were used. Because of embryonic lethality in *Srf*-null mice, we used conditional mutants of *Srf* that were obtained by crossing mice in which the *Srf* gene was flanked by loxP sites (*Srf* ^f/f^) with an CaMKCreER^T2^ line to ablate its expression exclusively in excitatory forebrain neurons [12, 21]. Additionally, to enable the time-specific induction of SRF deletion, adult mice (≥8 weeks old) were injected with tamoxifen, which stimulates the translocation of recombinant Cre recombinase to the nucleus. Eight weeks after mutation activation, homozygous adult *Srf* ^f/f^ mice (CTR) and *Srf* gene KO mice that carried a single copy of Cre recombinase *Srf* ^f/f;CaMKCreERT2^ (KO) were used for the experiments.

**Fig. 1:**
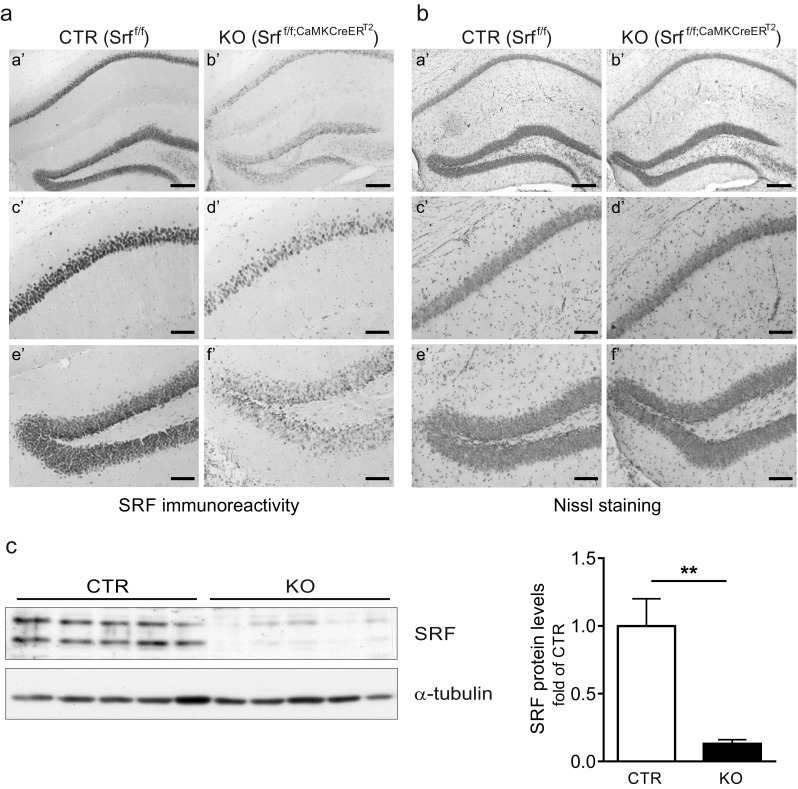
Conditional deletion of SRF in the adult hippocampus. **a** Immunohistochemical staining of SRF in the hippocampus in control (CTR; Srf^f/f^) and knockout (KO; Srf^f/f;CaMKCreERT2^) animals. Serum response factor elimination was observed in the dentate gyrus (DG) and CA1 subfield of the hippocampus. **b** Nissl staining of hippocampal sections from CTR and KO mice. No neuroanatomical differences were observed in the hippocampus in KO mice. Scale bar = **a**', **b**' 200 μm, **c**'–**f**' 400 μm. **c** Loss of two SRF isoforms (67 and 62 kDa) in protein extracts from DG in KO mice compared with CTR (Western blot and quantification of Western blot results), each *line* represents a single, independent animals, **p < 0.01 (Mann–Whitney test)

Immunohistochemical analysis of the brain slices obtained from KO animals (KO = *Srf* ^f/f;CaMKCreERT2^) and control littermates (CTR = *Srf* ^f/f^) revealed a specific decrease in SRF protein levels in the dentate gyrus (DG) and CA1 subfield of the hippocampus (Fig. 1a). Similar results that showed a large decrease in SRF protein levels in the DG in KO mice were obtained using Western blot (Fig. 1c). The remaining SRF staining may have resulted from its expression in astrocytes [29].

We found no evident neuroanatomical differences between CTR and KO animals’ brains. Nissl staining of hippocampal sections did not show any structural defects in KO animals (Fig. 1b). Similarly, the staining of nerve fibers with a zinc transporter member 3 (Znt3)-specific antibody revealed no significant differences in mossy fiber projections in CTR and KO adult brains (data not shown). Thus, the selective deletion of SRF in adult forebrain neurons did not cause general morphological abnormalities as a consequence of SRF deletion during the early stages of development [15, 14].

### SRF Deficiency Enhances Kainic Acid-Induced Epileptogenesis

To test whether SRF deficiency in the adult brain is sufficient to cause spontaneous aberrant neuronal activity, electroencephalograms (EEGs) were recorded from sham-operated, saline-injected animals (*n* = 6 per group, males). EEG activity was monitored daily for 18 days, 24 h/day, beginning 7 days after electrode implementation. EEGs were recorded from hippocampal and cortical electrodes. Sham-operated CTR and KO animals did not develop spontaneous seizures under basal conditions (data not shown). However, in all of the experiments performed in CTR and KO mice, three of 40 female KO mice developed seizures after i.p. saline injection, and this phenomenon was not observed in KO males and in CTR mice.

To study the phenotype of KO animals in the context of epileptogenesis, we used the kainic acid model of TLE. Because SRF KO animals were created on a C57BL/6 genetic background and mice with this genetic background do not show neurodegeneration upon systematic administration of kainic acid (data not shown and [30]), epilepsy was induced by an intrahippocampal injection of kainic acid [30]. To detect seizures, animals (*n* = 6 per group) were monitored by EEG video continuously for 18 days, 24 h/day, starting just after the intrahippocampal kainic acid administration. During the 18 days of recordings, all of the CTR and KO mice developed seizures.

**Fig. 2:**
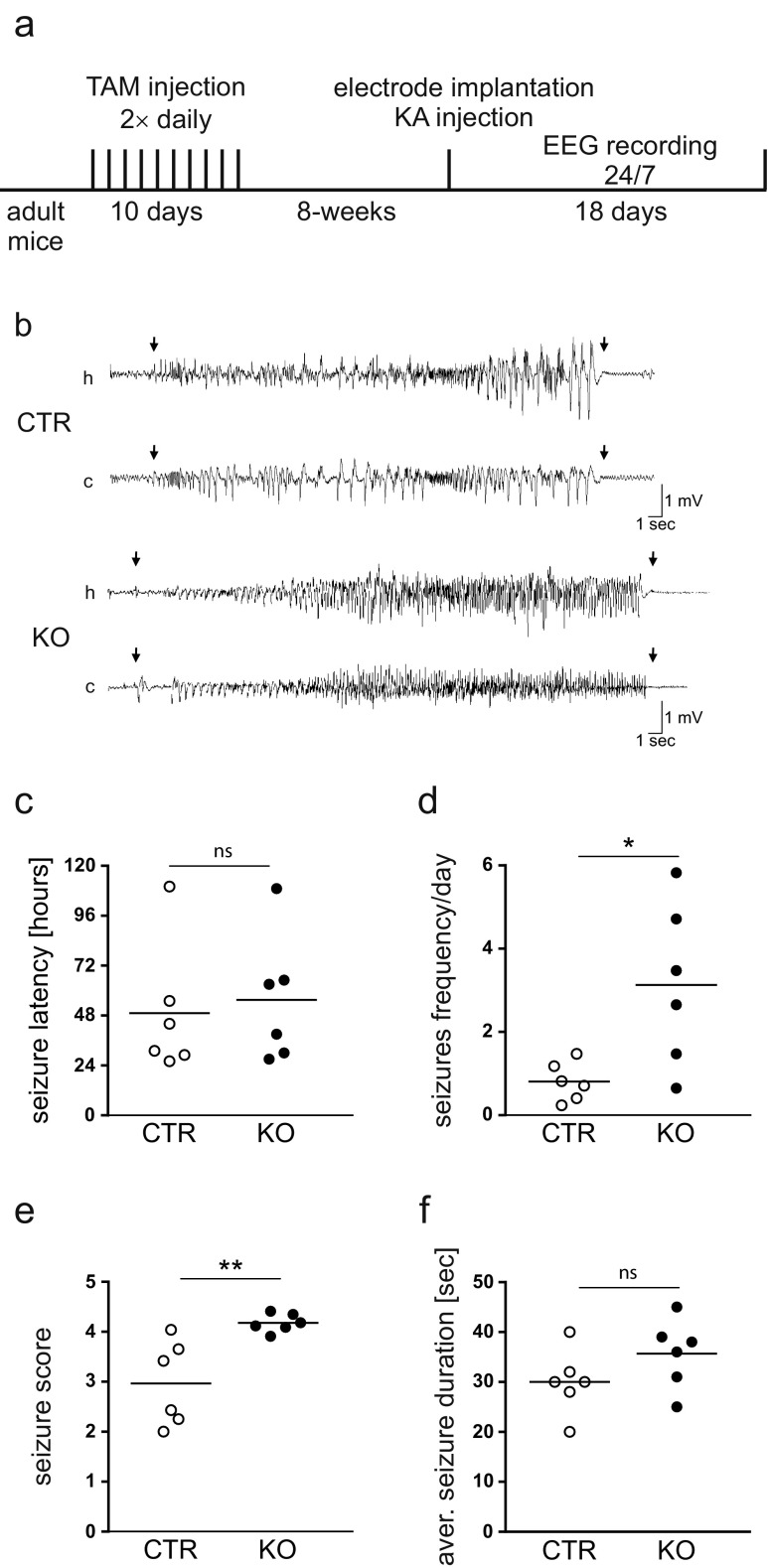
Lack of SRF increases number and duration of spontaneous seizures in the kainic acid model of TLE. **a** Schematic representation of the experimental design. **b** Representative EEG recordings of a spontaneous seizure in a CTR mouse and KO mouse, with a hippocampal (*h*) electrode and cortical (*c*) electrode (CTR, 5 days after intrahippocampal kainic acid injection, 33 s duration, behavioral severity on Racine scale = 3; KO, 6 days after intrahippocampal kainic acid injection, 36 s duration, behavioral severity on Racine scale = 5). The beginning and end of the seizures are indicated by *arrows*. **c** CTR and KO animals did not differ in the latency to the first spontaneous seizure. **d** Knockout animals developed a greater average number of seizures per day than CTR animals (Mann–Whitney test, *p* < 0.05). **e** Knockout animals showed more severe behavioral seizures measured according to Racine’s scale (Mann–Whitney test, *p* < 0.01). **f** The mean duration of spontaneous seizures was not significantly different in the KO group compared with the CTR group (Student’s *t* test, *p* > 0.05). All EEG recordings were conducted in males

During the first 24 h, we did not observe any differences in latency, number of seizures, seizure frequency, and seizures score (Supplementary Fig. S1). Also, the latency to the first spontaneous seizure did not significantly differ between KO and CTR animals (55.50 ± 12.57 h vs. 49.17 ± 12.95 h; Student’s *t* test, *p* > 0.05; Fig. 2c). Importantly, however, the seizure frequency was higher in KO animals than in CTR animals (3.13 ± 0.80 seizures/day vs. 0.81 ± 0.19 seizures/day, respectively; Mann–Whitney test, *p* < 0.05; Fig. 2d). The behavioral analysis of spontaneous seizures revealed that KO animals developed more severe seizures than CTR animals (Mann–Whitney test, *p* < 0.01; Fig. 2e). However, average spontaneous seizure duration did not significantly differ between KO and CTR animals (35.7 ± 2.8 vs. 30.0 ± 2.6 s, respectively; Student’s *t* test, *p* > 0.05; Fig. 2f). Similar epileptic phenotypes were observed for another cohort of animals (*n* = 12 per group) that were monitored by EEG video continuously for 7 days, 24 h/day, 10 days after kainic acid administration (data not shown). Altogether, our findings indicate that the lack of SRF-enhanced epileptogenesis.

### Loss of SRF Leads to Specific Deficits in Activity-Induced Gene Expression

**Fig. 3:**
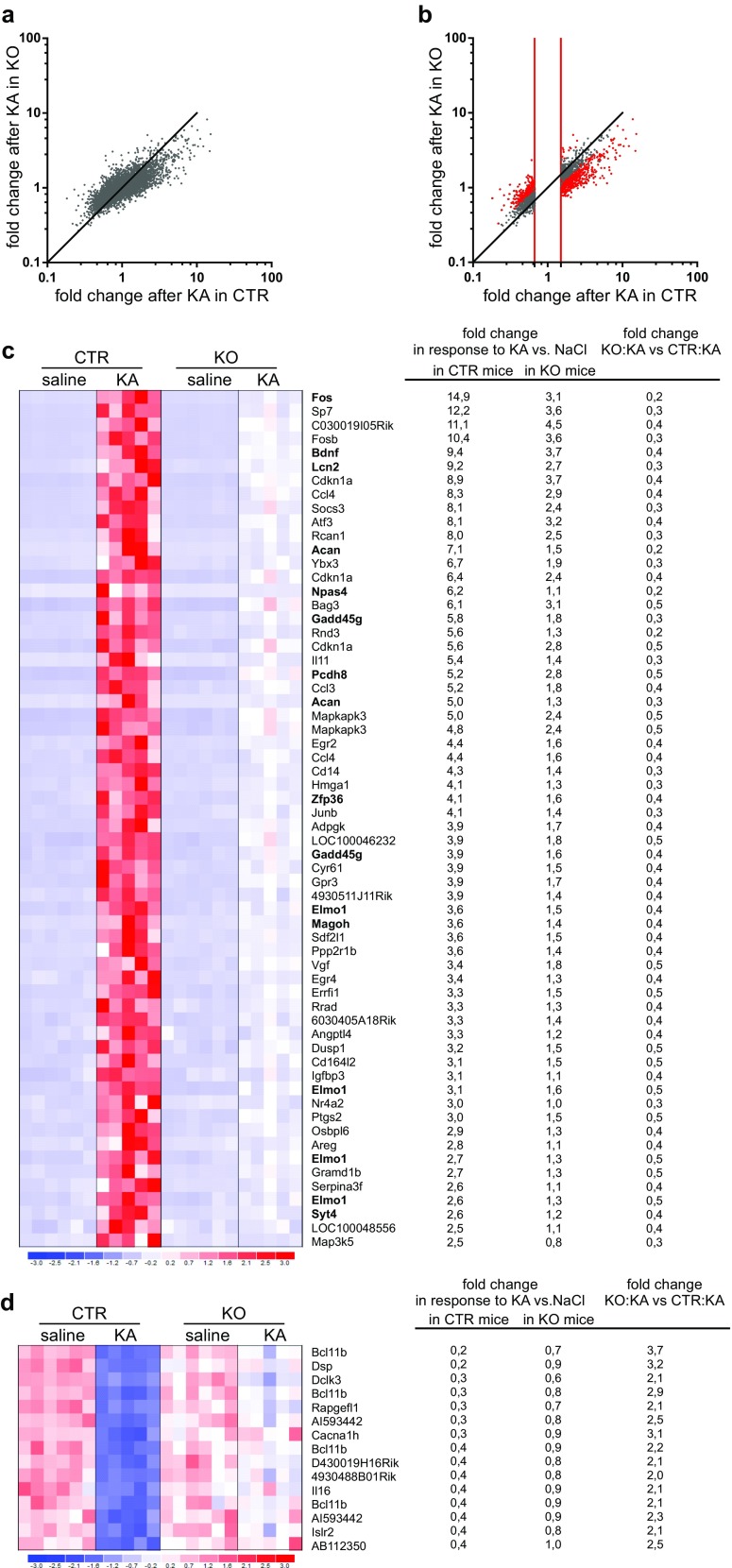
Microarray analysis shows that SRF is an important regulator of gene transcription in response to kainic acid-induced status epilepticus. The figure shows the results of gene expression profiling performed on RNA isolated from the dentate gyrus of the hippocampus in control (CTR) and Srf conditional knockout (KO) mice 6 h after kainic acid-induced status epilepticus (KA; intraperitoneal injection) or saline injection (saline). Twenty-two animals (males) were analyzed (5/6 animals per each genotype/treatment combination). **a** Scatter plot showing fold change values for KA responding genes in CTR and SRF mutants. **b** Pool of 3241 probes which expression was significantly changed at least 1.5 fold after KA (fold change > 1.5 or < 0.66; Tukey’s test, *p* < 0.05). *Red dots* indicate population of genes (729 probes) differentially regulated in SRF KO as compared to CTR (fold change CTR KA vs. KO KA >1.5 or <0.66 and Tukey’s test, *p* < 0.05). The *red lines* indicate microarray probes changed by kainic acid at least 1.5 fold. **c**, **d** In the heatmaps, *each column* corresponds to one animal with the indicated genotype/treatment combination. *Rows* represent transcripts as indicated on the right. *Colors* indicate normalized expression values as shown by the scale at the bottom (log2 change). The results for the 77 transcripts with the highest changes after kainic acid-induced status epilepticus in KO vs. CTR are shown (fold change in response to kainic acid >2.5 or <0.4, genotype × treatment interaction, FDR < 1 %; fold change in KO vs. CTR after kainic acid >1.96 or <0.51). Transcripts are ordered by fold change in CTR animals treated with kainic acid vs. saline. The values correspond to the indicated fold change (*linear scale*). The transcripts that were chosen for further analysis are shown in bold. **c** Selected genes upregulated in CTR animals in response to kainate but not changed or upregulated to a lesser extent in KO animals after kainic acid. **d** Genes downregulated in response to kainic acid in CTR animals but not changed or inhibited to a lesser extent in KO animals

To investigate the molecular mechanisms that underlie the consequences of SRF deletion, we sought to identify SRF-dependent activity-induced genes. Global gene expression induced by intraperitoneal injection of kainic acid was analyzed by microarrays. We did not observe significant differences in seizures severity and latency to seizures (score 4 or 5) in CTR vs. KO animals (Supplementary Fig. S2). Six hours after seizure induction (i.e., seizure severity = 5, based on a modified Racine scale) or saline injection, CTR and KO animals were sacrificed, and the DG was microdissected and used for RNA isolation. To identify SRF-regulated genes, we used Illumina MouseWG-6 v. 2.0 Expression BeadChips that include 45,281 transcripts. We focused our microarray analysis on the DG because (i) it is not vulnerable to kainic acid-induced excitotoxicity, (ii) it undergoes aberrant plasticity, and (iii) DG circuitry controls seizure propagation into the hippocampus [31, 32]. Kainic acid induces recurrent seizures, and the selected time point (i.e., 6 h) after the induction of status epilepticus enabled us to observe not only late-response genes but also early-response genes (see Fig. 3; e.g., *Fos*, *JunB*, *FosB*, *Egr2*, *Egr4*).

The statistical analysis of the microarray results showed no significantly downregulated transcripts under basal conditions (i.e., in saline-treated animals) in the DG in SRF KO mice, with the exception of *Srf* itself (Tukey’s test, *p* < 0.05; fold change < 0.66). Moreover, two transcripts (*Arsi* and *Sstr1*) were significantly upregulated in KO mice compared with CTR mice (Tukey’s test, *p* < 0.05; fold change > 1.5). This result was consistent with our EEG recording data, showing no difference in neuronal activity in CTR vs. KO animals under basal conditions (data not shown).

In contrast, comparisons of the gene expression profiles of the two genotypes (KO and CTR) in response to kainic acid-induced status epilepticus showed robust differences (Fig. 3a). Importantly, however, the loss of SRF in adult neurons did not cause general impairment in activity-dependent gene expression in the DG. Upon seizure induction, 3241 transcripts were significantly up- or downregulated in CTR animals (fold change > 1.5 or < 0.66; Tukey’s test, *p* < 0.05) (Fig. 3b). Among this group, only 729 probes were differentially regulated by KA in KO as compared to CTR (fold change > 1.5 or < 0.66; Tukey’s test, *p* < 0.05). The remaining 2512 probes (3241 − 729 = 2512; 77.5 %) did not meet the criteria (fold change CTR KA vs. KO KA: >1.5 or <0.66 and Tukey’s test, *p* < 0.05). Thus, SRF deficiency in adult neurons altered only a subset of genes, suggesting the specificity of the regulation. To further restrict our analysis and obtain a list of the most significantly changed transcripts, we performed a two-way analysis of variance (ANOVA) with false discover rate (FDR) correction at the 1 % level. Using this approach, we identified 431 probes (378 genes) with a significantly altered expression profile as a result of SRF knockdown after kainic acid (ANOVA, genotype × treatment interaction, *p* < 0.0005; correction FDR < 1 %; multiplicity of changes in CTR animals after the administration of kainic acid >1.5 or <0.66, Table S1).

Among those 378 genes, two main groups of genes were clearly distinguishable: (i) genes upregulated in CTR animals in response to kainic acid (216 genes) and not changed or upregulated to a lesser extent in KO animals after kainic acid and (ii) genes downregulated in response to kainic acid in CTR animals (162 genes) and not changed or inhibited to a lesser extent in KO animals after kainic acid. Genes with the most prominent differences in the expression profile between the two genotypes after kainic acid are shown in the heatmaps (Fig. 3a: 62 transcripts upregulated after kainic acid in CTR animals >2.5, fold change in KO vs. CTR after kainic acid < 0.51; Fig. 3b: 15 transcripts downregulated after kainic acid in CTR animals <0.4; fold change in KO vs. CTR after kainic acid >1.96). The probes are ordered by fold induction in CTR animals after kainic acid-induced status epilepticus. The genes that were chosen for further analysis (as described in the next paragraph; i.e., functions associated with the regulation of neuronal excitability and structural plasticity) are shown in bold. The values correspond to the indicated fold change (linear scale). To identify the overrepresentation of transcription factor binding sites (TFBSs) in the group of identified genes, we performed an in silico analysis using the cis-regulatory elements in the mammalian genome (cREMaG) database [23]. We found a significant overrepresentation of SRF binding sites in the group of 216 genes that were upregulated after kainic acid, suggesting the contribution of genes that are directly regulated by SRF (3.7-fold higher than expected by chance, *p* = 0.0194). Additionally, a significant overrepresentation of MEF2A, another MADS-box family transcription factor, was also found (3.7-fold higher than expected by chance, *p* = 0.0184).

### Functional Classification of SRF-Dependent Genes

To functionally classify the identified SRF-dependent transcripts, lists of downregulated and upregulated genes were analyzed by Gene Ontology (GO, DAVID). In the group of genes with decreased abundance in KO animals, the overrepresentation of transcripts that are involved in Behavior (4.1-fold enrichment, *p* = 3.48E^−6^; e.g., *Egr1*, *Egr2*, *Bdnf*, *Ntrk2*, *Cyr61*, *Nr4a2*, and *Nr4a3*) and MAPK signaling pathway (6.3-fold enrichment, *p* = 9.71E^−11^; e.g., *Ntrk2*, *Bdnf*, *Rps6ka3*, *Gadd45g*, *Map2k3*, *Mapkapk3*, and *Map3k5*) was observed.

To further extend the functional analysis, Ingenuity Pathway Analysis (IPA; Ingenuity® Systems, Qiagen) was applied. The Diseases and Bio-Functions analysis revealed Neurological Disease as a top-associated category (68 molecules; *p* = 4.55E^−25^ to 3.58E^−04^). The results of the analysis showed a significant correlation with annotations: Epilepsy (35 genes; *p* = 8.51E^−19^; Neurological Disease category; e.g., *Bdnf*, *Cacna1h*, *Fos*, *Gadd45g*, *Zfp36*, *Cyr61*, *Egr1*, *Egr2*, and *Egr4*), Plasticity of Synapse (9 genes; *p* = 0.000103; nervous system development and function category; e.g., *Bdnf*, *Ntrk2*, *Pcdh8*, and *Vgf*), and Outgrowth of Neurites (21 genes; *p* = 0.0000411; Nervous System Development and Function category; e.g., *Npas4*, *Bdnf*, *Ntrk2*, and *Gpr3*). In addition to GO and IPA, a manual analysis of gene function was performed, and their role in neurons was assigned based on published data (manual search in PubMed). Several functional groups were found, including (i) transcription factors and other regulatory proteins (e.g., *Fos*, *Npas4*, *Fosb*, *Junb*, *Egr1*, *Egr2*, *Egr4*, *Atf3*, *Sp7*, and *Cited2*) and (ii) genes with functions associated with the regulation of neuronal excitability and structural plasticity of dendritic spines (e.g., *Fos*, *Npas4*, *Bdnf*, *Syt4*, *Gadd45g*, *Acan*, *Lcn2*, *Pcdh8*, *Elmo1*, *Magoh*, and *Zfp36*; Table 3).

**Table 3:** SRF-dependent gene candidates that are important for the regulation of neuronal homeostasis. Summary and functional categorization of selected genes that represent potential candidates that may explain the SRF KO mouse phenotype of enhanced epileptogenesis. All of the genes are located in the deletion/duplication regions in human patients and are candidates for mental retardation or neurocognitive disabilities (based on DECIPHER database; http://decipher.sanger.ac.uk/; accessed July 7, 2014)

Gene symbol	Function	Neurological phenotype associated with deletion/duplication	Patient’s ID
*Fos*	IEG, regulator of neuronal excitability; expression induced in response to neuronal activity (e.g., after seizures); activated in human epileptic neocortex [75]; *Fos* KO mice have more severe kainic acid-induced seizures and increased neuronal excitability [70]	Intellectual disability	290148
*Npas4*	Transcription factor, IEG, selectively induced by Ca2^+^ influx; regulates homeostatic balance between excitation and inhibition in neurons by controlling the number of γ-aminobutyric acid-releasing synapses on excitatory neurons; *Npas4* KO animals are prone to seizures [58, 59]	Intellectual disability, delayed speech and language development	265913
*Bdnf*	Pro-plasticity neurotrophin, expression bi-directionally regulated by neuronal activity; regulates maturation and function of inhibitory synapses [33] as well as promotes synaptic transmission and synaptogenesis; activated in human epileptic neocortex [75]; lower serum BDNF levels were found in epileptic patients who suffer more frequent seizures [34]; in contrast, *Bdnf* KO mice display reduced epileptogenesis [35, 36], but conditional KOs do not have severely altered kindling [37]	Intellectual disability, delayed speech and language development	251208
*Syt4*	Upregulated in response to depolarization or seizures [38, 39]; modulates synaptic function by modulating BDNF release [62]; possible involvement in homeostatic plasticity; *Syt4* KO mice exhibit enhanced epileptiform responses [62]	Delayed speech and language development	250666
*Gadd45g*	Member of the GADD45 family associated with DNA damage repair and DNA demethylation; other members of GADD45 family (GADD45a and GADD45b) are involved in neurite outgrowth and activity-induced DNA demethylation (e.g., *Bdnf* and *Fgf*; [40, 63]); activated in human epileptic neocortex [75]	Autism, intellectual disability	270400
*Acan*	Component of perineuronal nets around parvalbumin interneurons; disruption of perineuronal nets leads to seizure-like activity in hippocampal cultures [72]; loss of aggrecan staining is observed after status epilepticus (1–2 weeks; [31])	Intellectual disability	261718
*Lcn2*	Small, inducible, secreted protein, identified as a protein associated with matrix metalloproteinase-9 [42]; *Lcn2* KO animals show increased spine density and neuronal excitability in hippocampus and amygdala [64, 74]	Autism, severe intellectual disability	289308
*Pcdh8*	Upregulated in response to neuronal activity or seizures; required for induction of long-term potentiation [43]; regulates dendritic spine number [65]; other members of Pcdhs family (*Pcdh10* and *Pcdh19*) are associated with neuropsychiatric disorders (epilepsy, mental retardation, autism-spectrum disorders; [44, 45]	Intellectual disability	260940
*Elmo1*	Regulates actin cytoskeleton reorganization; localized to excitatory synapses and is required for spine formation in hippocampal neurons [66]	Autism, severe intellectual disability	289704
*Magoh*	Core protein of the exon junction complex that regulates metabolism of spliced mRNA; targets mRNA for nonsense-mediated decay; controls brain size by regulating neural stem cell division [46]; lack of another exon junction complex component, eIF4A3, increases synaptic strength and GLUR1 AMPA receptor abundance at synapses and increases Arc protein levels [47]	Intellectual disability	272313
*Zfp36*	RNA-binding protein; interacts with AU-rich sequences in the 3′ untranslated region of targeted mRNAs and promotes their degradation; activated in human epileptic neocortex [75]	Global developmental delay	277936

The functional analysis of the group of SRF targets was consistent with the reported roles of SRF in the regulation of neurite outgrowth, synaptic plasticity, and behavior [15, 8, 12, 13, 48]. Thus, the identified SRF targets could explain the seizure-vulnerable phenotype observed in KO animals.

### Identification of SRF Direct Targets

**Fig. 4:**
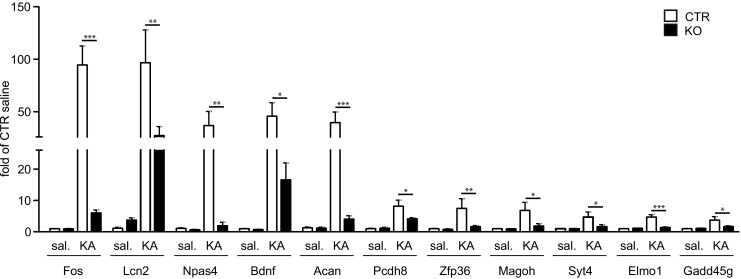
SRF is required for the activation of several plasticity genes in kainic acid-induced status epilepticus. The results obtained with microarray analysis were verified using qRT-PCR. qRT-PCR amplification of 11 transcripts from control (CTR) and knockout (KO) DG (saline, *n* ≥ 5 for each genotype; kainic acid, *n* ≥ 7 for each genotype; males and females) revealed an increase in mRNA levels in CTR animals in response to kainic acid stimulation (6 h after intraperitoneal injection of KA), which was abolished in KO animals. *p < 0.05, **p < 0.01, ***p < 0.001 (two-way ANOVA followed by Bonferroni post hoc test)

A group of genes with functions associated with the regulation of neuronal excitability and structural plasticity of spines was selected for validation. Microarray data were verified using quantitative real-time polymerase chain reaction (qRT-PCR; saline: *n* ≥ 5 for each genotype; kainic acid: *n* ≥ 7 for each genotype) for the selected transcripts. An increase in the expression level 6 h after kainic acid stimulation (intraperitoneal KA injection) and dependence on the transcription factor SRF was confirmed for FBJ osteosarcoma oncogene (*Fos*; *p* < 0.001), lipocalin 2 (*Lcn2*, *NGAL*; *p* < 0.01), neuronal PAS domain protein 4 (*Npas4*; *p* < 0.01), brain-derived neurotrophic factor (*Bdnf*; *p* < 0.05), *aggrecan* (*Acan*; *p* < 0.001), protocadherin 8 (*Pcdh8*; *p* < 0.05), zinc finger protein 36 (*Zfp36*; tristetraprolin [TTP]; *p* < 0.01), mago-nashi homolog, proliferation-associated (*Magoh*; *p* < 0.05), synaptotagmin IV (*Syt4*; *p* < 0.05), engulfment, and cell motility 1 (*Elmo1*; *p* < 0.001), and growth arrest and DNA-damage-inducible 45 gamma (*Gadd45g*; *p* < 0.05) two-way ANOVA genotype × treatment interaction, Bonferroni multiple-comparisons test (Fig. 4).

To determine which of the selected SRF-dependent genes are direct targets of SRF, we analyzed putative SRF binding sites within the evolutionary conserved regions between mouse and human, using two database tools, namely cREMaG [23] and NGD [24] (see “Materials and Methods” section for details). Additionally, we searched the human *CArGome* (according to the group of Miano; [27]) for potential CArG boxes that are conserved in mouse. Among the pool of potential SRF-binding sites identified with the above methods, only the motifs with a maximum of two mismatches to the CArG box consensus [CC(A/T)_6_GG] and with at most one mismatch in CC or GG were selected for the experimental validation.

**Fig. 5:**
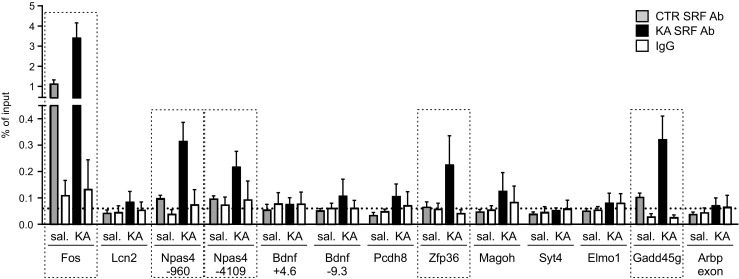
Endogenous SRF binds to the promoter regions of *Fos*, *Npas4*, *Gadd45g*, and *Zfp36*. The recruitment of the endogenous transcription factor SRF to the identified regions of selected genes was analyzed using chromatin immunoprecipitation with SRF-specific antibody, followed by qRT-PCR with primers surrounding predicted SRF-binding sites (for primer sequences and locations, see Table 2). Data are plotted as a percentage of input and are the averages of three independent experiments ± SEM. A fragment of *Arbp exon* was used as a negative control. The level of background was determined with normal IgG. Genes with SRF bound to their promoter fragments (*Fos*, *Npas4*, *Zfp36*, and *Gadd45g*) are marked with frames (>2-fold increase in precipitation level compared with *Arbp exon* and IgG controls). Kainic acid-induced status epilepticus (KA, intraperitoneal injection) enhanced the binding of SRF especially to the promoters of the *Npas4*, *Zfp36*, and *Gadd45g* genes. All ChIP analysis were conducted in males

To identify direct targets of SRF bound in vivo to the gene promoters in the hippocampus, we applied a model of kainic acid-induced status epilepticus. We investigated recruitment of the endogenous transcription factor SRF to the identified regions of selected genes using chromatin immunoprecipitation. Chromatin from the hippocampus in C57BL/6 mice that were treated with kainic acid (intraperitoneal kainic acid injection, 2 h after seizure onset) or naive mice was immunoprecipitated using an anti-SRF antibody or normal immunoglobulin G (IgG) to determine the background, followed by qRT-PCR amplification with specific primers (for the list of primers and potential CArG boxes, see Table 2). We observed the in vivo binding of SRF to the promoter of *Fos* under basal conditions (i.e., in naive animals), whereas a significant in vivo enrichment of SRF binding 2 h after seizure induction was observed for *Npas4*, *Gadd45g*, and *Zfp36* (Fig. 5). The binding of SRF to the promoters of those genes indicated direct regulation by SRF. They also presented a full CArG box consensus (or one mismatch in the case of *Zfp36*) in the identified SRE sequences. In contrast, we did not observe significant enrichment in SRF occupancy at potential CArG box regions of other analyzed genes (*Lcn2*, *Syt4*, *Bdnf*, *Magoh*, and *Pcdh8*). Altogether, we identified a group of novel SRF targets in neurons, which could explain the epilepsy-vulnerable phenotype observed in KO animals (Table 3). We showed that three of those genes (*Npas4*, *Gadd45g*, and *Zfp36*) are novel direct targets of SRF in the hippocampus in vivo, whereas the other identified genes are likely to be indirectly regulated by SRF in neurons.

## Discussion

The present study assessed the behavioral and transcriptional effects of SRF deficiency in neurons in the context of epileptogenesis. Animals with adult, neuronal deletion of SRF developed a more severe seizure phenotype compared with CTR animals in a kainic acid model of TLE. Using genome-wide analysis of activity-induced genes, we identified 378 genes that are differentially expressed in the hippocampal dentate gyrus of SRF-deficient mice, including regulators of inhibitory/excitatory balance and structural plasticity of neurons, together with three new SRF direct targets (*Npas4*, *Gadd45g*, *Zfp36*).

### SRF is an Important Transcription Factor in Epilepsy

Epilepsy is a clinically relevant form of hyperexcitability-associated brain pathology. Genes that are activated during seizures may contribute to epileptogenic processes via network reorganization that leads to hyperexcitability or via compensatory or protective mechanisms. In the present study, we uncovered SRF as an important transcription factor (TF) that regulates epileptogenesis. The deletion of SRF in the adult hippocampus increased the epileptic phenotype, manifested by more severe and frequent spontaneous seizures (Fig. 2), despite similar severity of acute seizures (Supplementary Fig. S1).

Activity-induced gene expression in neurons might also be regulated by the transcription factors CREB and MEF2. Despite partially overlapping patterns of gene expression, the inactivation of these TFs leads to divergent consequences. CREB deficiency decreases neuronal excitability and suppresses epileptogenesis [49–51]. MEF2 was shown to decrease the number of excitatory synapses and weaken neuronal strength [52, 53]. MEF2A binding sites, along with SREs, were identified in the present study as TF binding sites that were overrepresented in the promoters of genes regulated by SRF, suggesting that SRF and MEF2 can activate similar transcription programs in neurons.

### Possible Role of SRF-Dependent Genes in the Development of Epilepsy

Epilepsy is associated with robust synaptic plasticity that occurs at the cellular level caused by changes in gene expression [54]. Previous studies on SRF-dependent transcription in neurons focused mainly on basal gene expression or were performed using in vitro models [55, 56]. Although previous studies investigated the program downstream of SRF (recently reviewed by [57]), SRF-dependent transcription in neurons was focused mainly on a basal gene expression or were performed using in vitro models [55, 56]. SRF is one of the major regulators of plastic changes [12, 13]. This large-scale study of SRF-controlled transcription programs under in vivo conditions provides more insights into the molecular mechanisms that lead to the development of pathology. As expected, we found a large number of SRF-dependent genes associated with synaptic plasticity and epilepsy, the expression of which was decreased in SRF KO mice after seizures.

Functional annotation revealed that many of the SRF targets we have identified are known as regulators of inhibitory/excitatory balance, structural plasticity, and mRNA translation (see Table 3). Control of inhibitory/excitatory balance in neurons can be regulated by *Npas4*, which is an activity-dependent TF that controls inhibitory synapse development and a number of γ-aminobutyric acid-releasing synapses on excitatory neurons [58, 59]; *Bdnf*, which controls the maturation and function of inhibitory synapses [60, 61]; *Syt4*, which regulates synaptic function and plasticity by modulating BDNF release [62]; and *Gadd45g*, a member of the Gadd45 family that is engaged in the activity-induced demethylation of *Bdnf* promoters and transcription [63]. *Acan*, as a component of perineuronal nets around inhibitory interneurons, stabilizes synapses and restrict their reorganization.

Another group of identified genes are those encoding proteins regulating structural and physiological plasticity of excitatory neurons. *Lcn2*, *Pcdh8*, and *Elmo1* influence the electrophysiological properties of neurons by either decreasing dendritic spine density or changing their morphology [64–66]. These activity-induced proteins may suppress the number of spines to dampen synaptic function after elevated neuronal activity, similar to other neural activity-regulated molecules, such as MEF2 and PLK2 [53, 67].

A third group of genes are genes that encode RNA-binding proteins, such as *Magoh* and *Zfp36*, which regulate the metabolism of mRNAs by targeting them for degradation [68, 69].

The decreased production of proteins form any of mentioned above groups may explain the seizure-vulnerable phenotype observed in SRF KO animals. However, further studies are needed to address the specific role of SRF in the regulation of the balance between inhibition and activation in neurons.

We found an increase in epileptogenesis in SRF KO animals, which is consistent with the results of several studies that used animals with individual deletions of SRF-dependent genes. The ablation of such genes as *Npas4*, *Fos*, and *Arc* leads to increased seizure susceptibility [58, 70, 71]. The in vitro disruption of *Acan* causes seizure-like activity in hippocampal cultures [72]. Similarly, enhanced epileptiform responses were observed in slices from *Syt4* KO animals [73]. Moreover, *Lcn2*-deficient neurons show increased excitability [64, 74]. These proteins are induced by an increase in neuronal activity and appear to play a role as endogenous inhibitors of epilepsy; thus, impairment of their expression in SRF KO mice could enhance the epileptic phenotype, as demonstrated by the present results.

### SRF-Controlled Genes Associated with Human Pathology

Although no evidence for the role of SRF in human epilepsy was provided so far, it is important to note that either deletion or insertion in the regions of SRF target genes identified in our study was associated with human neurological disorders (Table 3). Single-nucleotide polymorphisms identified within or in close proximity to CArG boxes in humans were shown to be linked with neurological disorders, such as bipolar disorder, amyotrophic lateral sclerosis, and Alzheimer’s disease [27]. Several SRF-controlled genes that were identified in the present study (e.g., *Cyr61*, *Bdnf*, *Zfp36*, *Fos*, *JunB*) are upregulated in the cortex in patients who suffer from epilepsy. Statistically significant enrichment of SREs on the proximal promoters in this group of genes was observed [75].

In a group of genes that were downregulated in CTR animals after kainic acid-induced status epilepticus but not in KO animals (Fig. 3b), we identified *Cacna1h* (*Cav3.2*). Mutations in this gene are linked to a wide spectrum of idiopathic generalized epilepsies [76, 77] and influence neuronal excitability [78, 79]. The lack of downregulation of *Cacna1h* mRNA after status epilepticus in SRF KO mice may contribute to their enhanced epileptic phenotype.

### Molecular Mechanisms of SRF-Dependent Gene Regulation

Our results suggest that SRF can regulate gene expression by two possible molecular mechanisms. The first mechanism is direct binding to the gene promoters. We found in vivo enrichment of SRF occupancy on promoters of *Fos*, *Npas4*, *Gadd45g*, and *Zfp36*. Although *Fos* has been previously reported to be regulated by SRF, this is the first report of which we are aware on the direct regulation of *Npas4*, *Gadd45g*, and *Zfp36* by SRF in neurons. Significant enrichment of SRF binding to the new SRF targets was observed upon kainic acid stimulation in the present study. The binding of SRF to DNA can be constitutive, as observed for *Fos*, or inducible upon stimulation [80].

The second mechanism involves other transcription factors. Because of the relatively late time point analyzed in the microarray experiments (i.e., 6 h after seizure induction), SRF targets may be indirectly regulated through the activation of other genes that encode transcription factors (e.g., *Fos*, *Npas4*, *Egr1*), similar to our previous study that found that *Mmp*-*9* was regulated in neurons by SRF through Fos [81]. The identification of several transcription factors, among SRF target genes, that are important for neuronal plasticity suggests that SRF may be a primary hub that can orchestrate the regulation of several aspects of synaptic plasticity.

## Conclusions

Our data show that SRF is an important regulator of activity-induced gene expression in neurons and may be involved in the development of epilepsy. SRF regulates the expression of several plasticity genes that together may decrease hyperexcitation in response to a strong neuronal stimulation. The lack of these genes may lead to the development of a more severe TLE as a consequence of homeostatic imbalance. Still, further studies are needed to determine which of the identified SRF target genes are actually involved in the development of epilepsy and what is the molecular mechanism underlying this process. To address this question, identified genes need to be analyzed individually and their potential role in the context of epilepsy should be assessed.

## Electronic Supplementary Material

Below is the link to the electronic supplementary material.

Fig. S1 Severity of status epilepticus (SE) in CTR and KO animals after intrahipocampal kainic acid injection. CTR and KO animals did not significantly differ in latency to first induced seizure (a), number of seizure (b), mean duration of seizure (c) and seizure behavioral score (d) during first 24 hours after intrahippocampal kainate injection (a, b, c, d; Student’s *t*-test, *p* > 0.05). All EEG recordings were conducted in males (GIF 6 kb)

High resolution image (EPS 1649 kb)

Fig. S2 Severity of seizures in CTR and KO animals after intraperitoneal kainic acid injection. Results for CTR and KO animals did not significantly differ in seizure behavioral score (a) and latency behavioral seizure score 4 or 5 (b) after intraperitoneal administration of kainic acid. (a, b, Student’s *t*-test, *p* > 0.05). Analysis were conducted in males and females (GIF 4 kb)

High resolution image (EPS 955 kb)

Table S1 List of genes with a significantly altered expression profile as a result of SRF knockdown 6 hours after intraperitoneal kainic acid injections. (XLSX 117 kb)
