# Supplementary figures and images for: Adult Deletion of SRF Increases Epileptogenesis and Decreases Activity-Induced Gene Expression
Source: Mol Neurobiol. 2015 Jan 31;53(3):1478–93. doi: 10.1007/s12035-014-9089-7 (PMC4789231; doi:10.1007/s12035-014-9089-7)

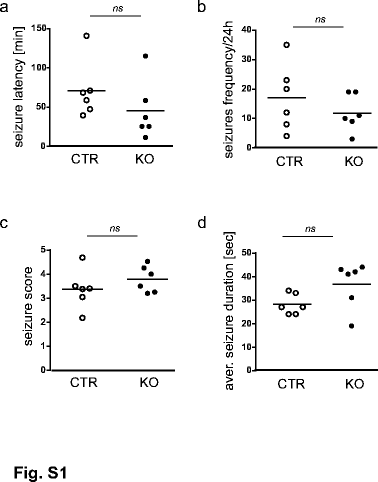

Supplement: Supplementary file 1 — Severity of status epilepticus (SE) in CTR and KO animals after intrahipocampal kainic acid injection. CTR and KO animals did not significantly differ in latency to first induced seizure (a), number of seizure (b), mean duration of seizure (c) and seizure behavioral score (d) during first 24 hours after intrahippocampal kainate injection (a, b, c, d; Student’s t-test, p > 0.05). All EEG recordings were conducted in males (GIF 6 kb) [file 12035_2014_9089_Fig6_ESM.gif]

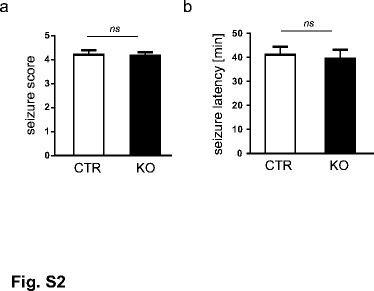

Supplement: Supplementary file 3 — Severity of seizures in CTR and KO animals after intraperitoneal kainic acid injection. Results for CTR and KO animals did not significantly differ in seizure behavioral score (a) and latency behavioral seizure score 4 or 5 (b) after intraperitoneal administration of kainic acid. (a, b, Student’s t-test, p > 0.05). Analysis were conducted in males and females (GIF 4 kb) [file 12035_2014_9089_Fig7_ESM.gif]
